# The Effect of ND:YAG Laser Posterior Capsulotomy Size on Refraction, Intraocular Pressure, and Macular Thickness

**DOI:** 10.1155/2014/846385

**Published:** 2014-03-03

**Authors:** Eyyup Karahan, Ibrahim Tuncer, Mehmet Ozgur Zengin

**Affiliations:** ^1^Alfagoz Eye Center, Mithatpasa Caddesi No. 247/A, Balcova, 35330 Izmir, Turkey; ^2^Department of Ophthalmology, Izmir University, Yeni Girne Bulvari 1825, Sokak No. 12, Karsiyaka, 35510 Izmir, Turkey

## Abstract

*Purpose*. The aim of this study is to examine the influence of capsulotomy size on, spherical equivalent (SE), intraocular pressure (IOP), and macular thickness. *Materials and Methods*. Sixty-eight patients were examined preoperatively and 1, 4, and 12 weeks after Nd:YAG capsulotomy. Patients were divided into two groups based on the postoperative capsulotomy size. Changes in SE, IOP, and macular thickness were compared between two groups. *Results*. We found a higher hyperopic shift in large capsulotomy group. In both groups 1 and 2, IOP increased 1 week postoperatively. Intraocular pressure rise in group 2 was higher than in group 1. Both groups had increased macular thickness at 1 week postoperatively. The degree of macular thickening was similar in group 1 and group 2. *Comment*. Patients who underwent a larger capsulotomy have a higher hyperopic shift and IOP elevation. Rise in macular thickness was similar in large and small capsulotomy groups.

## 1. Introduction

Introduction of sharp-edge optic intraocular lenses (IOL) and the development of the modern phacoemulsification technique have resulted in reduced rates of posterior capsule opacification (PCO) [1–3]. However, PCO is still the most common problem following cataract surgery [4]. Neodymium:yttrium-aluminum-garnet (Nd:YAG) laser capsulotomy is the standard treatment for PCO [5–7]. Although Nd:YAG laser capsulotomy has been found to be safe and effective, events such as retinal detachment [8–10], cystoid macular edema [10, 11], and rise in intraocular pressure (IOP) [12, 13] tend to occur after Nd:YAG laser capsulotomy.

Optical and mechanical factors should be considered for the optimal size of the posterior capsulotomy. Optical factors include diffraction, reduced image sensitivity, and glare. The optical considerations favor a large capsulotomy. Mechanical considerations are based on the barrier effect of the intact posterior capsule and favor a small capsulotomy [14]. Holladay et al. [14] concluded that the optimal capsulotomy should be equal or exceed the diameter of the pupil in the scotopic conditions and remain within the border of the IOL. The typical scotopic pupil diameter following extra capsular cataract extraction with a posterior chamber IOL varies between 3.9 mm and 5.0 mm [14–16].

The effect of capsulotomy size on refractive status after Nd:YAG laser procedure is contraversial. Findl et al. [17] have shown that large capsulotomy size is associated with increased posterior movement of the IOL. Theoretically, large capsulotomy size may cause a hyperopic shift. However, a previous study reported a refractive change of 0.38 diopters in the patients with capsulotomy size smaller than 4 mm and 0.22 diopters in the patients with capsulotomy size equal or larger than 4 mm [18]. Chua et al. [19] stated that spherical equivalent (SE) does not change after Nd:YAG laser capsulotomy.

Macular edema is caused by movement and damage in the vitreous cavity and release of inflammatory mediators due to the damage of blood-aqueous barrier; elevated IOP is associated with an increased amount of aqueous particles following Nd:YAG laser capsulotomy [20, 21]. Ari et al. [22] underlined that the severity and duration of increased IOP and macular thickness are less when a total energy level less than 80 mj is used. There is little information about the pure effect of capsulotomy size on IOP and macular thickness when Nd:YAG laser capsulotomy is performed with same energy levels.

The aim of this study was to examine the influence of Nd:YAG laser capsulotomy size on SE, IOP, and macular thickness.

## 2. Patients and Methods

This study was performed according to the tenets of the Declaration of Helsinki and written informed consent was obtained from either the patients or their legal guardians before enrollment. The study was a retrospective, observational study. A total of sixty-eight pseudophakic eyes of 68 patients with PCO following uncomplicated phacoemulsification with posterior chamber intraocular lens implantation surgery were included in the study. All patients were treated with Nd:YAG laser capsulotomy between January 2011 and February 2013 at a single center. The average time from cataract surgery was 32 months (range: 4–66). All patients were examined before Nd:YAG laser capsulotomy, 1, 4, and 12 weeks after Nd:YAG laser capsulotomy. All patients underwent a complete ocular examination on all visits, including best-corrected visual acuity (BCVA), refraction, slit-lamp, IOP measurement, and posterior segment examination. Objective refraction was measured using an autorefractometer (Nikon NRK 8000; Inc., Tokyo, Japan) after 20 minutes instillation of cyclopentolate 1% and tropicamide 1%. The spherical equivalent RE values were calculated as the sum of the sphere plus half the cylindrical power. All measurements were repeated 3 times. The average values were used in the analysis. The IOP was recorded by applanation tonometer. The OCT (Optuvue, Inc., Fremont, CA, USA) was administered for macular thickness measurements. Patients with corneal opacities, glaucoma, retinopathy, maculopathy, and optic neuropathy and patients with diabetes mellitus were excluded. Patients with high myopic and hyperopic refractive errors greater than −6.0 or +6.0 diopters were also excluded from this study.

Tropicamide 1% and phenylephrine 2.5% were administered for pupillary dilatation prior to procedure. After capsulotomy, prednisolone acetate 1% four times daily and apraclonidine hydrochloride 0.5% two times daily for 5 days were prescribed.

The capsulotomy size was determined with respect to scotopic pupil size. The scotopic pupil size was measured with slit-lamp and reticule. The purpose for capsulotomy size was to be equal or slightly exceed the diameter of the pupil in scotopic conditions. Patients were divided into two groups based on the postoperative capsulotomy size. The size of the capsulotomy was measured at 1 week after Nd:YAG laser capsulotomy. The size of the posterior capsule opening was measured vertically and horizontally using the reticule on the slit-lamp and the average of these values was accepted as capsulotomy size. Thirty-six patients with capsulotomy size smaller than 3.9 mm were accepted as group 1; thirty-two patients with capsulotomy size equal or larger than 3.9 mm were accepted as group 2. The total energy used in capsulotomy was 57.6 ± 8.9 mj (range: 42–72) in group 1 and 61.0 ± 12.7 mj (37–84) in group 2. There was no significant difference between two groups with respect to total energy (*P* = 0.214).

Two groups were compared based on changes in BCVA, SE, IOP, and macular thickness. Any serious anterior chamber reaction, cystoid macular edema, retinal tear, or detachment was also recorded.

SPSS, statistical software, version 11.6 (SPSS, Inc., Chicago, IL) was used for statistical analysis. The independent *t*-test was used for comparison of two groups and the paired *t*-test was used to detect intragroup differences for repeated measurements. Comparison of groups for gender was carried out with chi-square test.

## 3. Results

Mean age was 70.1 ± 7.8 years (range: 44–84) in group 1 and 71.5 ± 7.3 years (range: 56–91) in group 2. In group 1, 16 patients were male and 20 patients were female; in group 2, 17 patients were male and 15 patients were female. Mean age and gender were not significantly different between two groups (*P*: 0.478, 0.143, resp.)

Mean capsulotomy size was 3.43 ± 0.34 mm (range: 2.5–3.8) in group 1 and 4.56 ± 0.47 mm (range: 4.1–5.4) in group 2. Mean capsulotomy size was significantly larger in group 2 (*P*: 0.000).  Table 1 shows a comparison of two groups based on BCVA, SE, IOP, and macular thickness. No significant difference was found in BCVA, SE, IOP, and macular thickness between two groups.

Comparison of repeated measurements of BCVA, SE, IOP, and macular thickness in each group is shown in Tables 2 and 3. BCVA improvement at 1 week, 4 weeks, and 12 weeks was statistically significant in both small and large capsulotomy groups (*P* = 0.000). It was found that the SE decreased in the subsequent follow-up period in both groups. The hyperopic shift found in large capsulotomy group was higher than small capsulotomy group.

In both groups, IOP increased 1 week after Nd:YAG laser capsulotomy (*P* = 0.024, *P* = 0.001, resp.). Intraocular pressure rise in group 2 was higher than in group 1. Intraocular pressure declined to preoperative levels at 4 weeks after Nd:YAG laser capsulotomy in both groups. One patient (2.7 %) in group 1 and three patients (9.3%) in group 2 had mild elevation of IOP at 1 week after Nd:YAG laser capsulotomy. All of the patients with IOP elevation were treated successfully with antiglaucoma medications.

Both groups had increased macular thickness at 1 week after Nd:YAG laser capsulotomy. The degree of macular thickening was similar in group 1 and group 2. Mean macular thickness was decreased to preoperative levels at 4 weeks after Nd:YAG laser capsulotomy in both groups.

We did not observe any case of serious anterior chamber reaction and cystoids macular edema. However, a retinal tear in superior temporal quadrant was observed 4 weeks after Nd:YAG laser capsulotomy. This patient was treated with 360 degree laser photocoagulation with two rows around the retinal tear. The patient was followed with one week intervals. No subretinal fluid was examined around the retinal tear throughout the follow-up period.

## 4. Discussion

Posterior capsule opacification is the most common delayed complication of cataract surgery. The incidence of PCO was reported to be 20.7% at two years and 28.5% at 5 years after cataract surgery [16]. Although Nd:YAG laser capsulotomy has been found to be safe and effective, the procedure has potential to affect the position of the IOL. Findl et al. [17] reported that a subtle posterior shift of the posterior chamber IOL can occur but Thornval and Naeser [23] failed to observe this effect. In another study [19] the change in SE after Nd:YAG laser capsulotomy was statistically insignificant. Theoretically, posterior movement of the IOL may cause a hyperopic shift. In current study, we found a hyperopic shift in both small and large capsulotomy groups. The hyperopic shift was higher in large capsulotomy group than in small capsulotomy group. The hyperopic shift was progressive until 4 weeks in group 2. In light of our study, prescription of new spectacles should be deferred to at least 1 week or 4 weeks if the capsulotomy is large after Nd:YAG laser capsulotomy.

The most common complication of posterior capsulotomy is increased IOP. Despite the prophylactic treatment, increased IOP was reported in 15% to 30% of patients in several studies [24, 25]. Keates et al. [26] found elevation of IOP in 0, 6% of his patients, whereas Stark et al. [13] reported that the elevation of IOP was 1.0% after Nd:YAG capsulotomy. Ge et al. [27] found that rise in IOP was more pronounced in patients with glaucoma in those who experienced a higher rise of IOP within hour of capsulotomy. However, Shani et al. [28] could not find any elevation of IOP and postulated that healthy pseudophakic eyes do not generally show elevation of IOP after Nd:YAG laser capsulotomy. Ari et al. [22] also did not find any persistent rise in IOP. In our study, one patient (2.7%) in group 1 and three patients (9.3%) in group 2 had mild elevation of IOP one week after Nd:YAG laser capsulotomy. Rise in IOP was higher than previous studies. Previous studies did not give any information about the capsulotomy size. So, a comparison of rises in IOP with previous studies is not appropriate. All of the patients with IOP elevation were treated successfully with antiglaucomatous medication. Medical treatment was bringing to a successful conclusion within one month in all of the 4 patients. The mean IOP levels were significantly higher than preoperative levels in both groups. The rise of IOP was higher in large capsulotomy group when compared with small capsulotomy group. More capsule particles released with larger capsulotomies might be the reason of higher rates of elevation in group 2. We recommend using apraclonidine hydrochloride 0.5% two times daily for at least 5 days in either glaucomatous or nonglaucomatous patients.

One of the serious complications of Nd:YAG laser capsulotomy is that it leads to cystoid macular edema. Raza [29] reported cystoid macular edema in 3% of 550 patients treated with Nd:YAG laser capsulotomy for pseudophakic and aphakic posterior capsule opacification. This study was not designed to determine the effect of capsulotomy size or energy on cystoid macular edema after Nd:YAG capsulotomy. Ari et al. [22] evaluated how different energy levels of Nd:YAG laser capsulotomy affect macular thickness. They divided patients into two groups based on the energy levels used in Nd:YAG laser. They found that both groups had increased macular thickness compared to preoperative levels; macular thickness measurements of the patients treated with high energy levels were significantly higher compared to low energy levels. In another study a series of 897 Nd:YAG laser posterior capsulotomies were reviewed for the complications of cystoid macular edema. After Nd:YAG capsulotomy, 11 patients developed cystoid macular edema. The numbers of laser pulses and energy delivered were not risk factors [10]. Lewis et al. [30] studied 136 patients who underwent Nd:YAG laser capsulotomy for secondary opacification of the posterior capsule after extracapsular cataract extraction and they followed the patients for 6 months. Fluorescein angiography was repeated 4 to 8 weeks after the procedure. Cystoid macular edema did not develop in any of the patients in this series. In our study, energy levels were similar in both small sized and large sized capsulotomy groups. Comparison of two groups with respect to macular thickness did not reveal any difference preoperatively or 1 week, 4 weeks, or 12 weeks postoperatively. There was a significant thickening in macular thickness at 1 week in both groups 1 and 2; this difference was not statistically significant between groups. The mean macular thicknesses were decreased to preoperative levels at 4 and 12 weeks measurements in group 1 and group 2. In present study, any serious cystoid macular edema was not recorded during the follow-up period.

Retinal detachment is another serious complication after Nd:YAG laser capsulotomy. Raza [29] reported 11 patients (2%) of retinal detachment after Nd:YAG laser capsulotomy. Steinert et al. [10] reported that eight patients of 897 patients treated with Nd:YAG laser posterior capsulotomy developed retinal detachment. In our study, only one patient had retinal tear which was recognized 4 weeks after Nd:YAG laser capsulotomy. The SE was −3.25 diopters, Nd:YAG laser capsulotomy was administered 6 months after phacoemulsification, and capsulotomy size was 4.8 mm in this patient. There was no vitreous through the capsulotomy hole. The patient was examined with panendoscopic lens with one week intervals for development of retinal detachment. No subretinal fluid was seen during follow-up period. We recommend a detailed peripheral fundus examination after capsulotomy with regular intervals especially if the patient is myopic and the capsulotomy size is relatively large.

This study has limitations. The sample was small and represents the results at a single center only. The results were based on short-term follow-up period. Further follow-up might be required to determine the effect of capsulotomy size in Nd:YAG capsulotomy treatment. Another limitation of our study was that the exact mechanism of hyperopic shift after Nd:YAG laser capsulotomy did not appear in our study. We believe that examination of IOL movement with ultrasound biomicroscopy after Nd:YAG laser capsulotomy might be very helpful to determine the cause of differences in SE after Nd:YAG laser capsulotomy.

In conclusion, we found that patients who underwent a larger capsulotomy have a higher hyperopic shift and IOP elevation. Higher elevation of IOP in larger capsulotomy shows that the size of the Nd:YAG capsulotomy is a serious factor in Nd:YAG capsulotomy regardless of the used energy probably due to released inflammatory products. However, macular thickness was the same in large and small capsulotomy groups. Larger posterior capsulotomies may also lead to retinal tear and subsequent retinal detachment. Our treatment strategy and recommendation is individualizing to optimize the management of each particular case. Similar prospective trials will be needed to assess the effects of capsulotomy size on such variables investigated in our study.

## Figures and Tables

**Table 1:** Comparison of mean capsulotomy size, BCVA, SE, IOP, and macular thickness between groups before Nd:YAG laser capsulatomy and 1, 4, and 12 weeks after Nd:YAG laser capsulotomy.

	Group 1 (*n* = 36)	Group 2 (*n* = 32)	*P* value
Capsulotomy size	3.43 ± 0.34	4.56 ± 0.47	0.000*
BCVA (LogMAR)			
Pretreatment	0.61 ± 0.15	0.66 ± 0.18	0.209
1 week	0.18 ± 0.10	0.17 ± 0.13	0.776
4 weeks	0.18 ± 0.12	0.20 ± 0.12	0.637
12 weeks	0.21 ± 0.16	0.20 ± 0.12	0.834
SE (diopters)			
Pretreatment	−1.12 ± 1.23	−1.26 ± 1.26	0.630
1 week	−0.95 ± 1.09	−0.89 ± 1.06	0.841
4 weeks	−0.93 ± 1.04	−0.82 ± 1.02	0.662
12 weeks	−0.91 ± 1.05	−0.80 ± 1.05	0.663
IOP (mmHg)			
Pretreatment	15.44 ± 2.73	15.37 ± 3.61	0.929
1 week	16.33 ± 2.42	17.40 ± 3.26	0.126
4 weeks	15.13 ± 2.68	15.25 ± 3.95	0.892
12 weeks	14.83 ± 2.10	15.71 ± 2.30	0.102
Macular thickness (µm)			
Pretreatment	247.5 ± 31.3	244.5 ± 37.2	0.720
1 week	262.8 ± 27.2	259.9 ± 24.9	0.652
4 weeks	247.3 ± 36.8	246.7 ± 32.5	0.945
12 weeks	246.0 ± 29.6	242.9 ± 28.7	0.665

BCVA: best-corrected visual acuity; SE: spherical equivalent; IOP: intraocular pressure; LogMAR: logarithm of the minimum angle of resolution.

*Statistically significant.

**Table 2:** Comparison of repeated measurements of BCVA, SE, IOP, and macular thickness in group 1.

	Mean	*P* value
BCVA (LogMAR)		
1 week to pretreatment	−0.4 ± 0.2	0.000*
4 weeks to pretreatment	−0.4 ± 0.2	0.000*
12 weeks to pretreatment	−0.4 ± 0.2	0.000*
4 weeks to 1 week	0.02 ± 0.07	0.822
12 weeks to 4 weeks	0.02 ± 0.1	0.421
SE (diopters)		
1 week to pretreatment	0.16 ± 0.32	0.004*
4 weeks to pretreatment	0.18 ± 0.49	0.029*
12 weeks to pretreatment	0.23 ± 0.51	0.010*
4 weeks to 1 week	0.02 ± 0.26	0.638
12 weeks to 4 weeks	0.01 ± 0.23	0.720
IOP		
1 week to pretreatment	0.9 ± 2.2	0.024*
4 weeks to pretreatment	−0.3 ± 2.4	0.463
12 weeks to pretreatment	−0.6 ± 2.8	0.205
4 weeks to 1 week	−1.2 ± 1.5	0.000*
12 weeks to 4 weeks	−0.3 ± 2.0	0.384
Macular thickness		
1 week to pretreatment	15.2 ± 24.9	0.001*
4 weeks to pretreatment	−0.3 ± 19.5	0.926
12 weeks to pretreatment	−1.5 ± 17.9	0.606
4 weeks to 1 week	−15.5 ± 19.8	0.000*
12 weeks to 4 weeks	−1.2 ± 13.2	0.573

BCVA: best-corrected visual acuity; SE: spherical equivalent; IOP: intraocular pressure; LogMAR: logarithm of the minimum angle of resolution.

*Statistically significant.

**Table 3:** Comparison of repeated measurements of BCVA, SE, IOP, and macular thickness in group 2.

	Mean	*P* value
BCVA (LogMAR)		
1 week to pretreatment	−0.5 ± 0.2	0.000*
4 weeks to pretreatment	−0.5 ± 0.2	0.000*
12 weeks to pretreatment	−0.5 ± 0.2	0.000*
4 weeks to 1 week	0.2 ± 0.1	0.188
12 weeks to 4 weeks	0.03 ± 0.1	0.860
SE (diopters)		
1 week to pretreatment	0.36 ± 0.44	0.000*
4 weeks to pretreatment	0.44 ± 0.48	0.000*
12 weeks to pretreatment	0.46 ± 0.50	0.000*
4 weeks to 1 week	0.07 ± 0.13	0.002*
12 weeks to 4 weeks	0.01 ± 0.15	0.572
IOP		
1 week to pretreatment	2.0 ± 2.2	0.000*
4 weeks to pretreatment	−0.1 ± 2.1	0.750
12 weeks to pretreatment	−0.1 ± 3.1	0.822
4 weeks to 1 week	−2.1 ± 2.1	0.000*
12 weeks to 4 weeks	−0.4 ± 3.3	0.429
Macular thickness		
1 week to pretreatment	15.3 ± 21.1	0.000*
4 weeks to pretreatment	2.1 ± 16.7	0.484
12 weeks to pretreatment	−1.6 ± 20.8	0.657
4 weeks to 1 week	−13.3 ± 14.8	0.000*
12 weeks to 4 weeks	−3.8 ± 10.0	0.044*

BCVA: best-corrected visual acuity; SE: spherical equivalent; IOP: intraocular pressure; LogMAR: logarithm of the minimum angle of resolution.

*Statistically significant.
